# Estimating causal effects of genetically predicted type 2 diabetes on COVID-19 in the East Asian population

**DOI:** 10.3389/fendo.2022.1014882

**Published:** 2022-12-07

**Authors:** Masahiro Yoshikawa, Kensuke Asaba, Tomohiro Nakayama

**Affiliations:** ^1^ Division of Laboratory Medicine, Department of Pathology and Microbiology, Nihon University School of Medicine, Tokyo, Japan; ^2^ Technology Development of Disease Proteomics Division, Department of Pathology and Microbiology, Nihon University School of Medicine, Tokyo, Japan; ^3^ Department of Computational Diagnostic Radiology and Preventive Medicine, The University of Tokyo Hospital, Tokyo, Japan

**Keywords:** COVID-19, SARS-CoV-2, type 2 diabetes mellitus, Mendelian randomization, East Asia

## Abstract

**Background:**

Observational studies suggested that type 2 diabetes mellitus (T2DM) was associated with an increased risk of coronavirus disease 2019 (COVID-19). However, Mendelian randomization (MR) studies in the European population failed to find causal associations, partly because T2DM was pleiotropically associated with body mass index (BMI). We aimed to estimate the causal effects of T2DM on COVID-19 outcomes in the East Asian (EAS) population using a two-sample MR approach.

**Methods:**

We obtained summary statistics from a genome-wide association study (GWAS) that included 433,540 EAS participants as the exposure dataset for T2DM risk and from COVID-19 Host Genetics Initiative GWAS meta-analyses (round 7) of EAS ancestry as the outcome dataset for COVID-19 susceptibility (4,459 cases and 36,121 controls), hospitalization (2,882 cases and 31,200 controls), and severity (794 cases and 4,862 controls). As the main MR analysis, we performed the inverse variance weighted (IVW) method. Moreover, we conducted a series of sensitivity analyses, including IVW multivariable MR using summary statistics for BMI from a GWAS with 158,284 Japanese individuals as a covariate.

**Results:**

The IVW method showed that the risk of T2DM significantly increased the risk of COVID-19 susceptibility (odds ratio [OR] per log (OR) increase in T2DM, 1.11; 95% confidence interval [CI], 1.02–1.20; P = 0.014) and hospitalization (OR, 1.15; 95% CI, 1.04–1.26; P = 0.005), although the risk of severity was only suggestive. Moreover, IVW multivariable MR analysis indicated that the causal effects of T2DM on COVID-19 outcomes were independent of the effect of BMI.

**Conclusions:**

Our MR study indicated for the first time that genetically predicted T2DM is a risk factor for SARS-CoV-2 infection and hospitalized COVID-19 independent of obesity in the EAS population.

## Introduction

Coronavirus disease 2019 (COVID-19) was identified as atypical pneumonia cases that were caused by severe acute respiratory syndrome coronavirus 2 (SARS-CoV-2) in Wuhan, in the Hubei Province of China, in December 2019. Currently, COVID-19 has spread worldwide and is a major global burden, with 581,686,197 confirmed cases, including 6,410,961 deaths reported to the World Health Organization as of 8 August 2022 (1). The identification of risk factors for COVID-19 outcomes can lead to more effective prevention through medication and vaccination priorities for patients.

Observational studies suggested that type 2 diabetes mellitus (T2DM) was associated with an increased risk of SARS-CoV-2 infection and/or COVID-19 severity (2). Gao et al. reported observational associations of T2DM with COVID-19 outcomes after adjusting for body mass index (BMI) in a prospective cohort study, including 435,504 UK Biobank participants (hazard ratio [HR], 1.42; 95% confidence interval [CI], 1.24–1.63 for susceptibility; HR, 1.38; 95% CI, 1.11–1.70 for hospitalization; and HR, 1.65; 95% CI, 1.29–2.11 for death) (3). However, observational studies are generally prone to suffer bias due to potential confounders (4). Randomized controlled trials (RCTs) might be ideal but will be infeasible because it is ethically and practically impossible to randomize patients based on COVID-19 risk factors (5, 6). A two-sample Mendelian randomization (MR) study is a well-established method that mimics the design of an RCT using single nucleotide variants (SNVs, also known as single nucleotide polymorphisms [SNPs]) as instrumental variables (IVs) and can estimate a causal effect of a risk factor (exposure) on a trait of interest (outcome). As SNVs do not vary during an individual’s lifetime, the estimated effect by MR analysis can be interpreted as the lifelong effect of the genetically predicted risk factor (7). Because SNVs are randomly assigned and distributed at conception, MR studies are less likely to suffer from possible confounders and can overcome the limitations of observational studies (4). In fact, Gao et al. conducted MR analyses and revealed that the observational associations of T2DM with COVID-19 outcomes were totally mediated by the effect of BMI (3). Other MR studies also failed to find a causal effect of genetically predicted T2DM risk on COVID-19 outcomes in the European population (6, 8–12), partly because T2DM had a pleiotropic association with BMI. To our knowledge, all MR studies investigating associations between T2DM and COVID-19 were conducted in the populations of European or mostly European ancestry, and an MR study based on a genome-wide association study (GWAS) of non-European ancestry has been warranted (9, 12).

In the present study, we aimed to estimate causal effects of genetically predicted T2DM risk on risk of COVID-19 susceptibility, hospitalization, and severity that were independent of the effect of BMI using two-sample MR analyses in the East Asian (EAS) population.

## Materials and methods

All analyses were conducted using the TwoSampleMR package (version 0.5.6) in R software (version 4.0.3) (13). In the MR analyses, a P-value below 0.017 (0.05/3 by Bonferroni correction for multiple testing) was considered statistically significant, and a P-value between 0.017 and 0.05 was suggestively significant.

### Datasets

For the dataset of genetically predicted T2DM risk (binary trait), we obtained summary-level data from the EAS GWAS meta-analysis without adjustment for BMI (77,418 T2DM cases and 356,122 healthy controls) (14). The meta-analysis combined 20 GWAS cohorts and three biobanks (China Kadoorie Biobank, Korea Biobank Array, and Biobank Japan) in the EAS population. Each study established T2DM ascertainment criteria (14). In the China Kadoorie Biobank, for instance, eligible criteria were history of diabetes or fasting plasma glucose ≥7.0 mmol/L or non-fasting plasma glucose ≥11.1 mmol/L, and cases were excluded if diabetes was diagnosed under 35 years of age and insulin use was reported. The summary-level data was available from the MRC IEU Open GWAS database (15) (GWAS ID: ebi-a-GCST010118). For the datasets of genetically predicted COVID-19 risk (binary traits), we obtained summary-level GWAS data of EAS ancestry (4,459 cases and 36,121 population controls for COVID-19 susceptibility, 2,882 hospitalized COVID-19 cases and 31,200 population controls for COVID-19 hospitalization, and 794 very severe respiratory confirmed COVID-19 cases and 4,862 population controls for COVID-19 severity). Hospitalized COVID-19 cases were defined as patients hospitalized for laboratory-confirmed SARS-CoV-2 infection due to corona-related symptoms, and very severe respiratory confirmed COVID-19 cases were defined as patients hospitalized for laboratory-confirmed SARS-CoV-2 infection who died or were given respiratory support. Population controls consisted of genetically ancestry-matched individuals without known SARS-CoV-2 infection. The summary-level data were available from the COVID-19 Host Genetics Initiative (HGI) GWAS meta-analyses (round 7) (8, 16) (see Data availability statement). There was no obvious study overlap between T2DM and COVID-19 datasets. For the dataset of genetically predicted BMI (continuous trait), we obtained summary-level data from a GWAS in 158,284 Japanese individuals (17). The summary-level data was available from the MRC IEU Open GWAS database (15) (GWAS ID: bbj-a-1).

### Selection of IVs

From the exposure GWAS data, we selected SNVs as IVs that were associated with the exposure trait significantly (P <5.0 × 10^−8^) and independently (not in linkage disequilibrium [LD] [r^2^ <0.01 and distance >10,000 kb] with the other SNVs) using the clump_data function (population = “EAS”). We extracted summary statistics for each IV from both the exposure and outcome GWASs and harmonized them. We did not include proxy SNVs in the analyses. We excluded palindromic SNVs with an intermediate minor allele frequency >0.42 according to the default settings of the harmonise_data function. To evaluate the strength of the IVs, we calculated the F-statistic for each SNV (18, 19). IVs with an F-statistic below 10 are considered weak instruments (20).

### Two-sample MR and sensitive analyses

We conducted a two-sample univariable MR study to estimate the causal effects of T2DM on COVID-19 using genetically predicted T2DM risk as an exposure and genetically predicted risk of COVID-19 susceptibility, hospitalization, and severity as outcomes. The Wald ratio estimates the causal effect for each IV as the ratio of beta for the corresponding SNV in the outcome dataset divided by beta for the same SNV in the exposure dataset (21). Beta means effect estimate (SD unit) per allele for BMI (a continuous trait) or log odds ratio (OR) increase per allele for T2DM and COVID-19 (binary traits). As a main analysis of this MR study, we conducted a meta-analysis of each Wald ratio using the inverse variance weighted (IVW) method. We presented the results as the OR of COVID-19 risk per log (OR) increase in T2DM by exponentiating the combined estimate.

IVs must satisfy the following three assumptions: the IVs are associated with the exposure (IV assumption 1); the IVs affect the outcome only *via* the exposure (IV assumption 2); and the IVs are not associated with measured or unmeasured confounders (IV assumption 3). IV assumption 1 was satisfied by selecting SNVs as IVs that were associated with the exposure trait at P <5.0 × 10^−8^. However, IV assumptions 2 and 3 can be violated by horizontal pleiotropy and confounders (21), and it is difficult to satisfy IV assumptions 2 and 3 strictly (22). Therefore, we conducted a series of sensitivity analyses, including Cochran’s Q statistic calculation, the MR-Egger regression method, the weighted median method, the weighted mode method, the MR-PRESSO global and outlier tests, the leave-one-out sensitivity analysis, and the IVW multivariable MR analysis (21). Cochran’s Q statistic with the corresponding P-value measures heterogeneity among the causal estimates across all the SNVs in the IVW method. If heterogeneity is low, the causal estimate is more reliable (23). When Cochran’s Q statistic was significant (P <0.05), we used a multiplicative random-effects IVW model as high heterogeneity indicated potential pleiotropy of the included IVs; otherwise, we used a fixed-effects model. The MR-Egger regression intercept differs from zero with statistical significance (P <0.05) when IV assumption 2 is violated and horizontal pleiotropy occurs (24). The weighted median method provides a valid estimate if more than half of the IVs satisfy the IV assumptions (24). The weighted mode method clusters the SNVs based on similarity of causal effects and provides an estimate from the largest cluster (21). A leave-one-out sensitivity analysis was conducted by removing each SNV from the IVW analysis and re-estimating the causal effect. If the effect changes drastically, there is a possibility that the IVW result is driven and biased by a single outlying SNV with a large horizontal pleiotropic effect (21, 24). The MR-PRESSO global and outlier tests were performed using the run_mr_presso function to detect possible horizontal pleiotropy and potential outlier SNVs at P <0.05 (25). IVW multivariable MR analysis was conducted using the mv_ ivw function to estimate the direct effects of genetically predicted T2DM risk on COVID-19 outcomes adjusted for the effects of genetically predicted BMI (26, 27).

## Results

**Figure 1:**
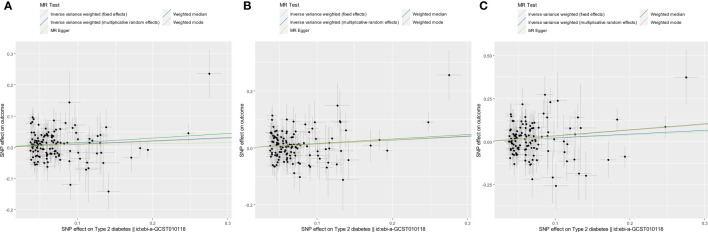
Scatter plots for estimating the causal effects of genetically predicted T2DM risk on the risk of COVID-19 susceptibility **(A)**, hospitalization **(B)**, and severity **(C)**. Each black point representing the size of the effect size of each SNP on the exposure (horizontal axis) and the outcome (vertical axis) is plotted with error bars corresponding to each SE. Each effect size is given as a log (OR) increase per allele. The slope of each line corresponds to the combined estimate using each method: the fixed-effects IVW (light blue line), the multiplicative random-effects IVW (blue line), the MR-Egger regression (light green line), the weighted median (green line), and the weighted mode (red line). The slopes of the light blue line and the blue line were quite the same. Each OR was calculated by exponentiating each of the slopes. For example, the slope of the IVW line was 0.10, and the OR of COVID-19 susceptibility per log (OR) increase in T2DM was 1.11 **(A)**.

First, we conducted univariable MR analyses to estimate the causal effects of genetically predicted T2DM risk on the risk of COVID-19 outcomes. Of the 174 instrumental SNVs that were associated with T2D risk at P <5.0 × 10^−8^, 33 were removed due to LD with other SNVs or absence from the LD reference panel in the EAS population. A total of 10 instrumental SNVs were not identified in the COVID-19 severity dataset. Moreover, 13, 14, and 14 SNVs were removed for being palindromic with intermediate allele frequencies after harmonizing, leaving a total of 128, 127, and 117 instrumental SNVs in the COVID-19 susceptibility, hospitalization, and severity datasets, respectively (**Supplementary Table 1**). The F-statistics of every instrumental SNV were >10, indicating there was no weak instrumental bias. The univariable MR results are shown in **Table 1**, **Figure 1**, and **Supplementary Figures 1**–**3**. The fixed-effects IVW method showed that the risk of T2DM significantly increased the risk of COVID-19 susceptibility (OR per log (OR) increase in T2DM, 1.11; 95% confidence interval [CI], 1.02–1.20; P = 0.014) and COVID-19 hospitalization (OR, 1.15; 95% CI, 1.04–1.26; P = 0.005). From sensitivity analyses, other MR methods also provided directionally consistent results. Cochran’s Q statistic for the IVW method (P >0.05) indicated low heterogeneity and indicated reliability of the causal effect. MR-Egger intercept (P >0.05) and MR-PRESSO global test (P >0.05) suggested a lack of possible horizontal pleiotropy. Leave-one-out sensitivity analysis for COVID-19 susceptibility showed that the significance disappeared when each of the two SNVs was excluded from the IVW method (**Supplementary Figure 1**); however, the MR-PRESSO outlier test revealed that there were no outlier SNVs. Because Cochran’s Q statistic for the IVW method (P <0.05) indicated heterogeneity for COVID-19 severity, we conducted the random-effects IVW method, which suggested that the risk of T2DM increased the risk of COVID-19 severity (OR, 1.24; 95% CI, 1.02–1.52; P = 0.035). However, the MR-PRESSO global test (P <0.05) indicated possible horizontal pleiotropy.

**Table 1:** MR results of the effect of T2DM on COVID-19 risk.

Exposure traits	Outcome traits	Number of SNVs	IVW method	MR-Egger regression method		Weighted median method	Weighted mode method	Heterogeneity (IVW)	MR-PRESSO global test
			OR (95% CI) *P*-value	OR (95% CI) *P*-value	Intercept(SE) *P*-value	OR (95% CI) *P*-value	OR (95% CI) *P*-value	Cochran’s Q *P*-value	*P*-value
Type 2 diabetes	COVID-19 susceptibility	128	1.11 (1.02–1.20) 0.014	1.03 (0.86–1.24) 0.75	0.006 (0.007) 0.39	1.16 (1.01–1.33) 0.04	1.10 (0.94–1.29) 0.23	152.0 0.06	0.07
Type 2 diabetes	COVID-19 hospitalization	127	1.15 (1.04–1.26) 0.005	1.14 (0.92–1.41) 0.23	0.0004 (0.008) 0.96	1.17 (0.99–1.38) 0.07	1.15 (0.93–1.44) 0.20	141.4 0.16	0.16
Type 2 diabetes	COVID-19 severity	117	1.24 (1.02–1.52) 0.035	1.20 (0.79–1.82) 0.38	0.0027 (0.016) 0.87	1.41 (0.98–2.03) 0.06	1.39 (0.93–2.09) 0.11	143.8 0.04	0.04

The IVW method showed that the OR of COVID-19 susceptibility, hospitalization, and severity per log (OR) increase in T2DM were 1.11 (95% CI, 1.02–1.20), 1.15 (1.04–1.26), and 1.24 (1.02–1.52), respectively. CI, confidence interval; COVID-19, coronavirus disease 2019; IVW, inverse variance weighted; MR, Mendelian randomization; OR, odds ratio; SE, standard error; SNVs, single nucleotide variants; T2DM, type 2 diabetes mellitus.

Next, we conducted IVW multivariable MR analyses to estimate the direct causal effect of genetically predicted T2DM risk on the risk of COVID-19 outcomes adjusted for the genetically predicted BMI effect. The IVW multivariable MR results are shown in **Table 2**. We obtained comparable results with univariable MR analyses regarding risk of COVID-19 susceptibility (OR per log (OR) increase in T2DM, 1.14; 95% CI, 1.04–1.25; P = 0.007), COVID-19 hospitalization (OR, 1.20; 95% CI, 1.08–1.35; P = 0.001), and COVID-19 severity (OR, 1.28; 95% CI, 1.03–1.59; P = 0.027), indicating that genetically predicted T2DM risk increased COVID-19 risk independently of BMI.

**Table 2:** IVW multivariable MR for the effects of T2DM on COVID-19 risk adjusted for BMI.

Exposure traits	Adjustment	Outcome traits					
		COVID-19 susceptibility		COVID-19 hospitalization		COVID-19 severity	
		Number of SNVs	OR (95% CI) *P*-value	Number of SNVs	OR (95% CI) *P*-value	Number of SNVs	OR (95% CI) *P*-value
Type 2 diabetes	BMI	150	1.14 (1.04–1.25) 0.007	149	1.20 (1.08–1.35) 0.001	136	1.28 (1.03–1.59) 0.027

The IVW multivariable MR method showed that the BMI-adjusted OR of COVID-19 susceptibility, hospitalization, and severity per log (OR) increase in T2DM were 1.14 (95% CI, 1.04–1.25), 1.15 (1.08–1.35), and 1.28 (1.03–1.59), respectively. BMI, body mass index; CI, confidence interval; COVID-19, coronavirus disease 2019; IVW, inverse variance weighted; MR, Mendelian randomization; OR, odds ratio; SE, standard error; SNVs, single nucleotide variants; T2DM, type 2 diabetes mellitus.

## Discussion

It remains controversial whether T2DM is a risk factor for COVID-19 severity and susceptibility. Most MR studies failed to show significant effects of genetically predicted T2DM risk on COVID-19 susceptibility, hospitalization, or severity using COVID-19 HGI round 3 to 6 data of European or mostly European ancestry (3, 6, 8–12, 28, 29). COVID-19 HGI showed significant genetic correlations between T2DM and COVID-19 outcomes using their round 5 data on European ancestry. Their univariable MR analyses also suggested a causal relationship between genetically predicted T2DM risk and severe COVID-19 (OR, 1.1; 95% CI, 1–1.2; P = 0.036); however, the MR result was not significant after being corrected for multiple testing (8). They inferred that the observed genetic correlations were due to pleiotropic effects between T2DM and BMI. Recently, COVID-19 HGI found significant causal association between T2DM risk and COVID-19 outcomes of European ancestry by univariable MR analyses using their round 6 data after correcting for multiple testing (OR, 1.02; 95% CI, 1.01–1.03; P = 0.0016 for susceptibility. OR, 1.06; 95% CI, 1.03–1.1; P = 0.00014 for hospitalization); however, the observed associations were diminished by IVW multivariable MR analyses using genetically predicted BMI as a covariate (29). Thus, the causal effect of genetically predicted T2DM risk on COVID-19 outcomes was suggested, if any existed, to be totally mediated by the effect of BMI in the European population. Otherwise, the effect could not be evaluated correctly in the case that younger subjects with genetically predicted risk in MR studies may not have developed T2DM (11, 12).

Our MR analyses indicated for the first time that genetically predicted T2DM risk significantly increased the risk of COVID-19 hospitalization and susceptibility independently of the effect of BMI in the EAS population. The differences from MR results in the European population may be due to interethnic differences in T2DM pathophysiology and epidemiology (30). EAS patients developed T2DM at a much lower BMI than European patients (31), and there was a weaker association between BMI and T2DM risk in Asians than in Caucasians (32). Moreover, EAS patients tended to develop young-onset T2DM at a younger age compared with European patients (30). These interethnic differences may explain the discrepancy between MR results in the EAS and European populations.

The underlying biological mechanisms by which T2DM increases SARS-CoV-2 infection and COVID-19 hospitalization remain unclear. Patients with diabetes were susceptible to other coronaviruses, including MERS-CoV (the Middle East respiratory syndrome coronavirus) and SARS-CoV-1 (33). Innate immunity, the first line of defense against infection, can be significantly altered by hyperglycemia, which inhibits neutrophil chemotaxis, phagocytosis, and superoxide production (34). A phenome-wide MR study using T2DM GWAS data of European ancestry and expression quantitative trait loci data in the lung of mostly European ancestry reported that T2DM was causally associated with increased gene expression of ACE2, a cellular entry receptor for SARS-CoV-2, which may increase COVID-19 susceptibility (35). A pooled analysis of observational studies found that COVID-19 patients with diabetes had significantly higher levels of inflammatory and hypercoagulability markers such as interleukin-6 (IL-6) and fibrinogen than non-diabetic COVID-19 patients (36), reflecting cytokine storm and intravascular coagulation that may eventually lead to disease progression in diabetic COVID-19 patients. Consistently, RCTs showed that IL-6 receptor antagonists improved COVID-19 outcomes, including survival (37, 38). Insulin resistance is also involved in hypercoagulability due to endothelial dysfunction and platelet aggregation, leading to pulmonary embolism and stroke (33).

Several limitations should be noted in the present study. First, as T2DM ascertainment criteria differ across the studies in the GWAS meta-analysis (14), cases of type 1 diabetes mellitus and maturity-onset diabetes of the young may be included. Second, our leave-one-out analysis showed that the result for COVID-19 susceptibility might be biased by possible horizontal pleiotropy. Although our MR-Egger intercept and MR-PRESSO test indicated little evidence of horizontal pleiotropy, the result needs to be interpreted carefully. Third, our univariable MR analyses using the IVW method for COVID-19 severity only suggested the causal effect of T2DM, and the MR-PRESSO global test (P <0.05) indicated possible horizontal pleiotropy. Moreover, the lifelong effects estimated by our MR study (e.g., OR per log (OR) increase in T2DM, 1.15; 95% CI, 1.04–1.26 for hospitalized COVID-19) may be rather small compared to the short-term effects estimated by observational studies (2, 3). Further studies using GWASs with a larger sample size in the EAS population might be able to overcome these limitations.

In summary, our MR study indicates that T2DM is a risk factor for SARS-CoV-2 infection and hospitalized COVID-19 independent of obesity in the EAS population. This is probably because diabetic patients are younger and less obese than those in the European population. It is suggested that diabetic patients should be prioritized for COVID-19 prevention, including SARS-CoV-2 vaccination, even if they are not obese.

## Data availability statement

The exposure GWAS dataset was publicly available from the MRC IEU Open GWAS database (https://gwas.mrcieu.ac.uk/datasets/) given as GWAS-IDs of “ebi-a-GCST010118” for T2DM risk, and “bbj-a-1” for BMI, respectively. The outcome GWAS dataset was publicly available from the COVID-19-HGI GWAS meta-analyses (round 7) (https://www.covid19hg.org/results/r7/ and https://www.covid19hg.org/covidhgi-freeze-7-readme.txt).

## Ethics statement

Ethical review and approval and written informed consent for participation were not required for this study because we used only publicly available GWAS summary-level datasets.

## Author contributions

MY designed this study, analyzed data, and wrote the draft of the manuscript. TN and KA discussed and reviewed the manuscript critically. All authors contributed to the article and approved the submitted version.

## Supplementary material

Supplementary Figure 1 Leave-one-out sensitivity analysis for estimating causal effect of T2DM on the risk of COVID-19 susceptibility. Each black point represents the combined causal estimates on the outcome per increase in the exposure using IVW methods with horizontal lines denoting 95% CI after removing the corresponding SNV from the analysis.

Supplementary Figure 2 Leave-one-out sensitivity analysis for estimating causal effect of T2DM on the risk of COVID-19 hospitalization.

Supplementary Figure 3 Leave-one-out sensitivity analysis for estimating causal effect of T2DM on the risk of COVID-19 severity.
