# Supplementary figures and images for: Estimating causal effects of genetically predicted type 2 diabetes on COVID-19 in the East Asian population
Source: Front Endocrinol (Lausanne). 2022 Dec 7;13:1014882. doi: 10.3389/fendo.2022.1014882 (PMC9767950; doi:10.3389/fendo.2022.1014882)

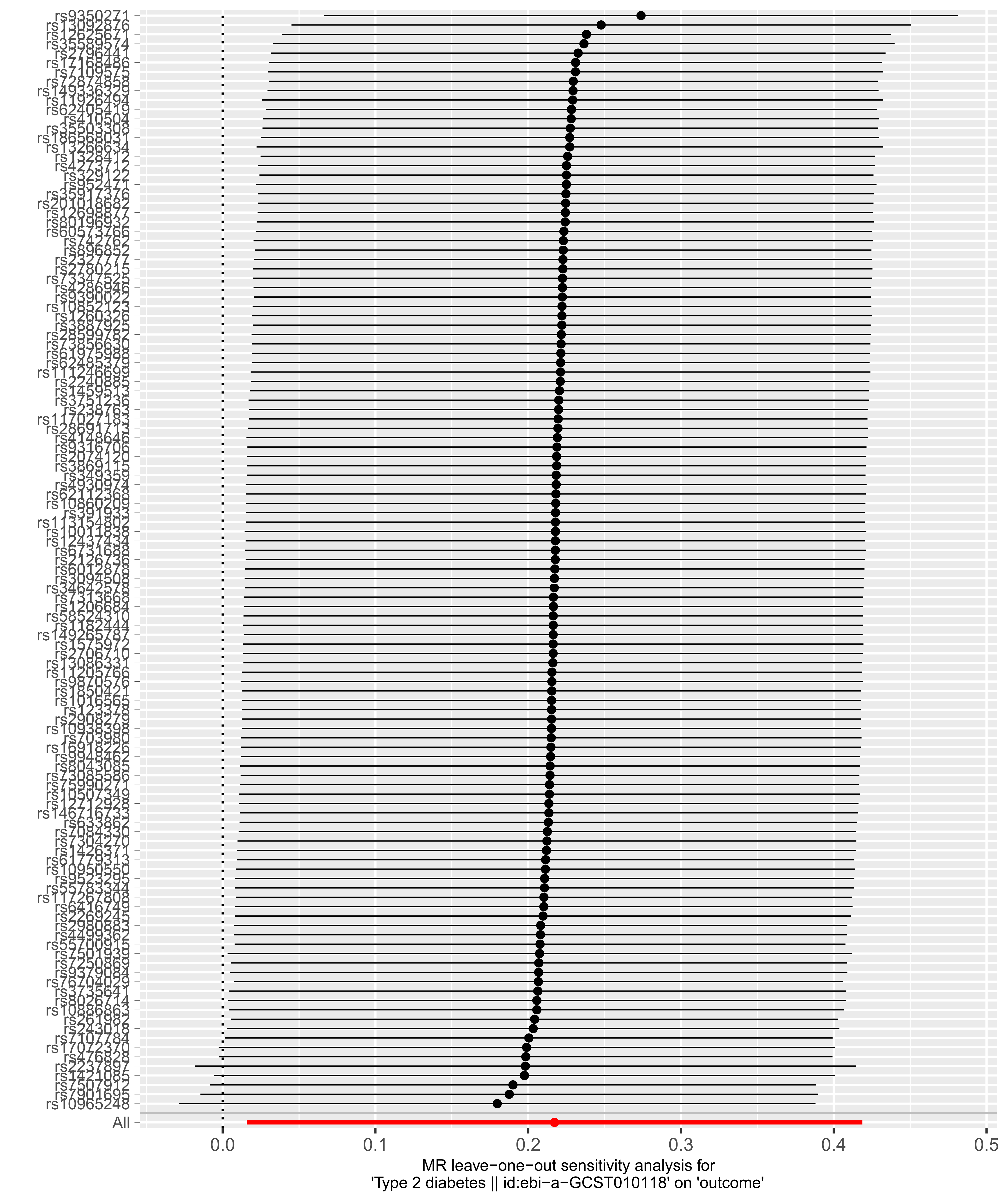

Supplement: Supplementary Figure 3 — Leave-one-out sensitivity analysis for estimating causal effect of T2DM on the risk of COVID-19 severity. [file Image_3.tif]
